# Homing of adipose stem cells on the human amniotic membrane as a scaffold: A histological study

**DOI:** 10.18502/ijrm.v18i1.6193

**Published:** 2020-01-27

**Authors:** Hooman Zarei, Abbasali Karimpour, Ali Reza Khalatbary, Fereshteh Talebpour Amiri

**Affiliations:** ^1^Department of Anatomy, Faculty of Medicine, Molecular and Cell Biology Research Center, Mazandaran University of Medical Sciences, Sari, Iran.; ^2^Student Research Committee, Faculty of Medicine, Mazandaran University of Medical Sciences, Sari, Iran.

**Keywords:** Amniotic membrane, Scaffold, Chondrogenesis, Differentiation, Mesenchymal stem cell.

## Abstract

**Background:**

The human amniotic membrane (HAM) is a suitable and effective scaffold for cell culture and delivery, and adipose-derived stem cells (ADSCs) are an important source of stem cells for transplantation and chondrogenic differentiation.

**Objective:**

To assess the practicability of a cryopreserved HAM as a scaffold in cell proliferation and differentiation in vitro.

**Materials and Methods:**

In this experimental study, adipose tissue samples were harvested from the inguinal region of male patients aged 15-30 years. Flow cytometry was used to identify CD31, CD45, CD90, and CD105 markers in adipose stem cells. HAM was harvested from donor placenta after cesarean section, washed, trypsin-based decellularized trypsinized decellularized, and used as a scaffold via three methods: 1) ADSCs were differentiated into chondrocytes on cell culture flasks (monolayer method), and after 14 days of culture, the cells were transferred and cultured on both sides of the HAM; 2) ADSCs were cultured and differentiated directly on both sides of the HAM for 14 days (scaffold-mediated differentiation); and 3) chondrocytes were differentiated with micromass culture for 14 days, transferred on HAM, and tissue slides were histologically analyzed qualitatively.

**Results:**

Flow cytometry confirmed the presence of mesenchymal stem cells. Histological findings revealed that the cells adhered and grew well on the stromal layer of HAM. Among the three methods, scaffold-mediated differentiation of ADSCs showed the best results.

**Conclusion:**

ADSCs have excellent attachment, viability, and differentiation capacity in the stromal side of HAM. Additionally, the direct culture and differentiation of ADSCs on HAM is more suitable than the culture of differentiated cells on HAM.

## 1. Introduction

Approximately 22.7% of the population of the United States has osteoarthritis and approximately 9.8% have arthritis-attributable activity limitation; these figures are expected to continuously increase with age (1). Due to the low repair capacity in articular cartilage and the lack of successful techniques for its repair, arthroplasty remains the main treatment for severe osteoarthritis. Its outcomes include pain reduction and mobility improvement for performance of activities of daily living (2). Furthermore, the most common reasons for total joint replacement are aseptic and septic loosening, dislocation, periprosthetic fractures, pain, wear, and technical errors, which require subsequent surgeries (3). Due to high surgical cost and limited effectiveness, the construction of a tissue-engineered cartilage may result in remarkable improvement in ADL performance of patients with osteoarthritis. The articular cartilage is considered a good source of endogenous chondrocytes, but the use of this type of cells is associated with further damage to the articular cartilage (4). Tissue engineering is a prospective approach suitable for the reconstruction of damaged cartilage (5). In cartilage tissue engineering involves a scaffold. seed cells, and growth factors (6). Owing to the limited use of autologous cells, researchers use allogeneic stem cells to prepare functional researchers use allogeneic stem cells for using in tissue engineering applications. In addition, because of the possible immunological rejection of allogeneic cells, the use of mesenchymal stem cells (MSCs) is important (7). Furthermore, the use of a decellularized extracellular matrix (ECM) as a scaffold to construct tissue-engineered cartilage has drawn considerable attention in recent years (8). Human adipose-derived stem cells (ADSCs), which can be easily obtained from subcutaneous fat tissues by liposuction or arthroscopy, are a suitable source of multipotent MSCs. In vitro expansion of these cells has shown that these cells remain undifferentiated and have no change in telomerase activity even after the ninth passage (9). Moreover, the chondrogenic differentiation capacity of ADSCs has been demonstrated in several studies (10). These characteristics as well as high proliferation potential, secretion of angiogenic factors and healing-associated growth factors, and easy culture and differentiation into other cell lines make ADSCs a promising cell source for tissue engineering and cell therapy (11). The human amniotic membrane (HAM) is a thin fetal tissue with favorable characteristics such as affordability, ease of availability, as well as antibacterial and anti-inflammatory properties. In addition, the presence of collagen types I, III, and IV; laminin; fibronectin; and various growth factors makes it a highly promising scaffold for cartilage tissue engineering (11). Cellular and acellular HAMs have been widely used clinically in regenerative medicine and/or experimental studies (12). In previous studies, fibroblasts (13), keratinocytes (14), epithelial cells (15), and chondrocytes (16) were cultured on amniotic membranes. In this study, for the first time, we cultured and differentiated ADSCs on HAM as a scaffold via three different methods.

The aim of this study was to investigate whether HAM could support the proliferation, attachment, and chondrogenic differentiation of ADSCs using histological evaluation.

## 2. Materials and Methods

### Stem cell isolation and culture

In this cross-sectional study, adipose tissue samples were obtained from the inguinal region of 15 male patients (age: 15-30 yr) who were referred for hernia repair. These samples were transferred to the cell culture lab in a Falcon tube containing PBS with 1% Pen/Strep. Explant culture was used to isolate ADSCs. In brief, samples were cut into small pieces (1-2 mm${}^{2}$) after washing twice with sterile PBS containing 1% Pen/Strep to remove blood and extra tissues. The tissue pieces were cultured in Petri dishes and were left undisturbed to allow the exit of cells from the margins of explants. After the cells grew out of the explants, the tissue pieces were removed and cells were passaged when they reached approximately 70% confluency. Third- or fourth-passage ADSCs were used for chondrogenic differentiation.

### Flow cytometry 

Flow cytometry was performed to confirm and identify the surface markers of MSCs. To this end, two specific surface markers of stromal MSCs (CD105 and CD90) and two specific markers of hematopoietic MSCs (CD31 and CD45) were studied. In brief, 2 × 10${}^{5}$ fourth-passage cells were transferred to each control and test Falcon tube after counting using a hemocytometer. Then, they were centrifuged for 5 min at 2500 rpm and the supernatant was drained. Cell deposition was solved in 3% BSA and incubated on ice for 30 min. Then, CD90, CD45, CD31, and CD105 conjugated with phycoerythrin (PE) antibodies were added to the test tubes. The samples were incubated for 1 h in dark at room temperature. Next, PBS was added to the tubes and centrifuged for 1 min at 2500 rpm. The supernatant was drained, and the labeled cell masses were dissolved in PBS and analyzed by flow cytometry (Becton Dickinson).

### HAM preparation

HAM was immediately isolated from the donor placenta using sterile scissors. Samples were washed with normal saline solution to remove blood; then, the samples were placed in a Falcon tube containing sterile PBS with 1% Pen/Strep and quickly transferred to the cell culture room of the anatomical laboratory (Mazandaran University of Medical Sciences, Iran). Next, under a laminar flow hood, the samples were washed twice with sterile PBS containing 1% Pen/Strep. For acellular HAM, trypsinization and freezing/defreezing (3 times) were additionally performed. Finally, HAM was transferred to a Falcon tube containing sterile PBS and stored at -18°C until use.

### Cell culture and chondrogenic differentiation using HAM as a scaffold

Culture and differentiation of ADSCs using HAM as a scaffold was performed using three methods:

• In the first method, 2.5 × 10${}^{5}$ fourth-passage ADSCs were first transferred to a 6-well culture plate. A chondrogenic differentiation medium (Invitrogen) was added and changed every 2 days. After 14 days, chondrogenic differentiated ADSCs were mechanically detached from the bottom of the wells with a cell scraper and transferred onto HAM.

• In the second method, HAM was loaded onto the bottom of a 6-well culture plate. Then, 2.5 × 10${}^{5}$ ADSCs were transferred to the center of HAM and a chondrogenic differentiation medium was added, which was changed every 2 days for 14 days.

• Micromass culture was used as the third method. After trypsinization and centrifugation, cells were resuspended in a small amount of chondrogenic differentiation medium to make a high-density cell solution containing 2.5 × 10${}^{5}$ cells/25 μL droplet. A 25 μL droplet was carefully placed at the center of every well of a 24-well culture plate. After 2-hr incubation, the chondrogenic differentiation medium was added to each well. The medium was changed every 3 days for a total of 2 wk.

DMEM containing 10% FBS and 1% Pen/Strep was used for the control group in all methods.

**Figure 1:**
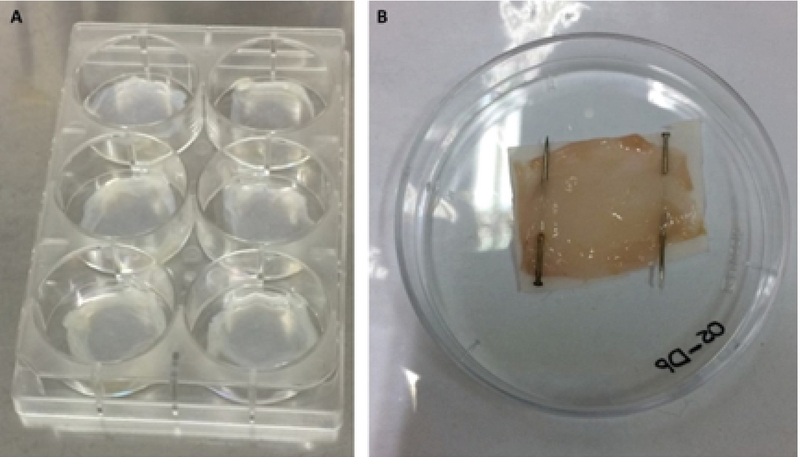
A) Human amniotic membrane attached to the bottom of a 6-well culture plate. B) Placing the sample on a filter paper using office pins for processing and embedding in paraffin.

To ensure adherence of HAM to the bottom of wells and to culture cells, HAM was cut into square pieces and fitted to the bottom of 6-well plates. Samples were transferred into the wells from both stromal and epithelial stromal and epithelial sides of HAM (Figure 1A). These pieces of HAM were incubated with 500 λ FBS for 1 hr before using them for homing and differentiation of the cells.

### Histological assessment

At the end of the culture and differentiation periods, HAM samples containing ADSCs and chondrogenic cells were fixed with 10% formalin for 24 hr. HAM samples containing chondrogenic cells and ADSCs were placed on filter paper and fixed using office pins (Figure 1B). The samples were dehydrated in the graded alcohols, embedded in paraffin, and stained sections and 5 micro meter thick sections stained by hematoxylin and eosin. All slides were examined by a histologist blindly under an optical microscope (Nikon).

### Ethical consideration

The instructions of the Ethics Committee of Mazandaran University of Medical Sciences were followed (IR.MAZUMS.REC.1393.1402), and informed consent was obtained from the patients admitted to Imam Khomeini Hospital in Sari.

## 3. Results

### Morphology of isolated cells 

**Figure 2:**
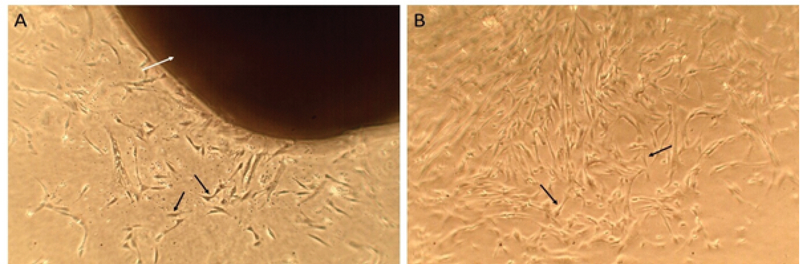
A) Heterogeneous cell population containing fibroblast-like cells (black arrows) that grew out from the margins of the tissue fragment (white arrow) were seen 7 days after adipose tissue fragments were explanted. B) The tissue fragment was removed at day 11 as the cells became confluent. Mag: ×400, scale bar: 100 μm.

Heterogeneous adherent cells were observed 7 days after explanted adipose tissue fragments were added to the flasks. This heterogeneous cell population comprised circulating blood cells, fibroblasts, pericytes, endothelial cells, and adipocyte progenitors (ADSCs). At this time, the adipose tissue fragments were removed from the flasks and the cells became confluent on day 11 (Figure 2). After trypsinization and subculture, the isolated ADSCs formed a homogenous population of adherent fibroblast-like cells.

### Flow cytometry

**Figure 3:**
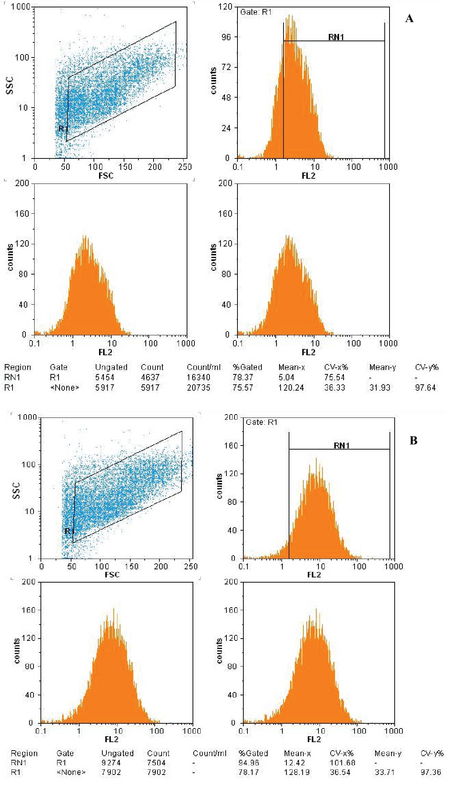

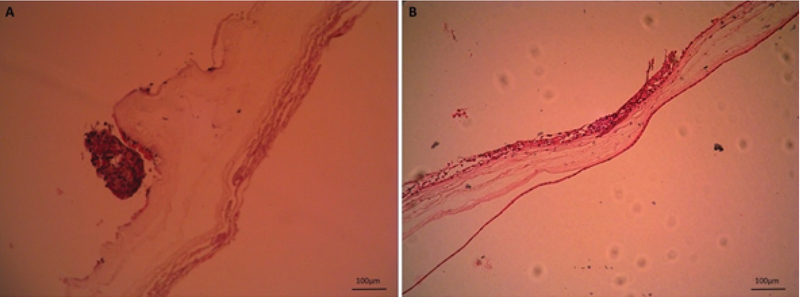
Immunophenotypic profile of adipose-derived stem cells (ADSCs). The mesenchymal stem cell (MSC) markers CD105 (A) and CD90 (B) were positive for ADSCs, whereas the hematopoietic MSC markers CD45 (C) and CD31 (D) were negative.

The analysis of MSC CD markers by flow cytometry showed that fourth-passage ADSCs expressed an average of 78.3% CD105 (Figure 3A) and 94.9% CD90 (Figure 3B), which are prominent MSC markers. However, <2% of these cells expressed CD45 (Figure 3C) and CD31 (Figure 3D).

### Histological findings

**Figure 4:**
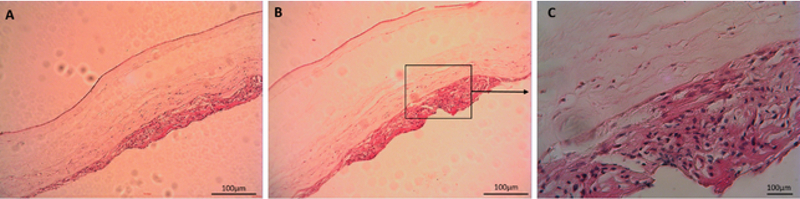
Adipose-derived stem cells did not adhere to the epithelial side (A), but they attached and integrated into the stromal layer of human amniotic membrane (B) (hematoxylin and eosin staining; Mag: ×10, scale bar: 100 μm).

As the first approach in this study, ADSCs were cultured on the epithelial and stromal sides of HAM. As shown in Figure 4A, ADSCs were seen separated from the epithelial side, but in Figure 4B, the cells were seen to be attached and proliferated. Additionally, some ADSCs had integrated into the stromal layer of HAM.

**Figure 5:**
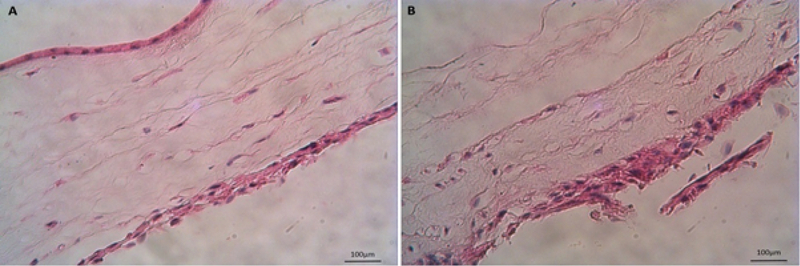
Culturing and differentiating adipose-derived stem cells on human amniotic membrane. (A) Control group, (B) differentiation group, and (C) selected area under high magnification (hematoxylin and eosin staining. A and B, Mag: ×100 and C, Mag: ×400; scale bar: 100 μm).

The next approach was to compare culturing and differentiating ADSCs directly and indirectly on HAM. In the first method, ADSCs were cultured and differentiated appropriately on the stromal side of HAM, and a proper thickness of chondrogenic cells was seen. However, in the control group, good proliferation was observed that was comparable to that in the differentiation group; and also, ADSCs integrated more into HAM (Figure 5).

**Figure 6:**
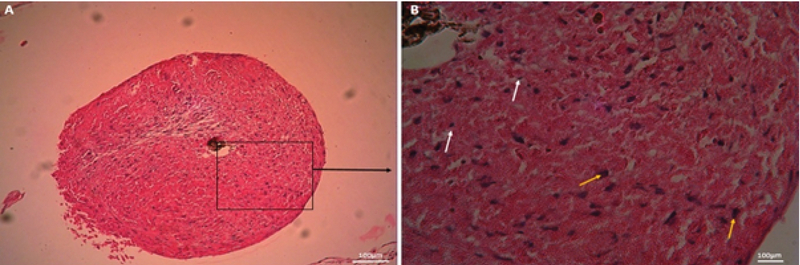
Culturing chondrogenic differentiated cells on human amniotic membrane. (A) Control group and (B) differentiation group (hematoxylin and eosin staining. Mag: ×400, scale bar: 100 μm).

In the second method, ADSCs were cultured and differentiated on a plate and then transferred from the plate to HAM. In this way, ADSCs completely adhered to the stromal side or surface of HAM, but compared with the previous method, their thickness was less. In the control group, ADSCs showed more integration into HAM (Figure 6).

**Figure 7:**
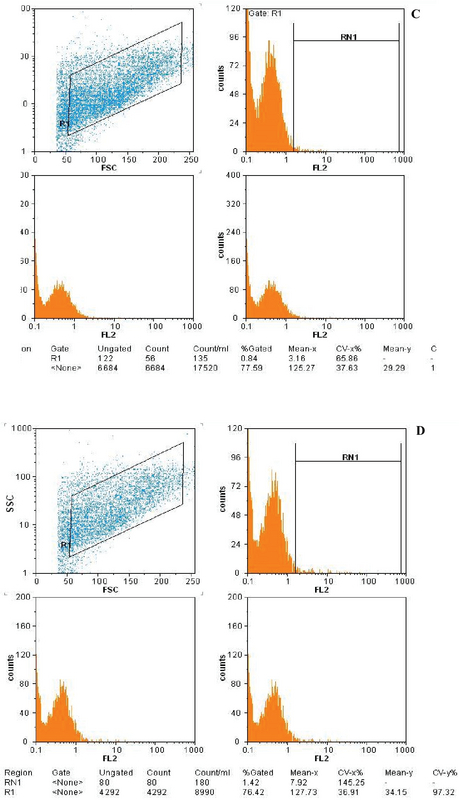
Micromass culture. (A) Low magnification and (B) high magnification of selected area. White arrows indicate necrotic cells, yellow arrows indicate differentiated cells (hematoxylin and eosin staining. Mag: A, ×100 and B, ×400; scale bar: 100 μm).

Regarding micromass culture, chondrogenic micromass was not appropriately attached to HAM, and necrotic cells were observed at the center of the micromass; however, in the peripheral portion, there were differentiated cells with a round nucleus (Figure 7).

## 4. Discussion

In this study, we cultured and differentiated ADSCs using HAM as a scaffold by three methods. We showed that ADSCs express MSC markers and that HAM is a suitable scaffold for cultivation and ADSC differentiation. We also demonstrated that the stromal side of HAM is more appropriate than the epithelial side and that scaffold-mediated differentiation is better than monolayer method and micromass culture. Moreover, the differentiation of ADSCs directly on HAM is more appropriate than the transfer of chondrogenic differentiated cells to HAM.

The ease of isolation and culture, high ability to differentiate into other Cell types, and high proliferation capacity make ADSCs a favorable source of stem cells for tissue engineering and cell therapy (11). These cells do not have the disadvantages of chondrocytes including restricted sources, high allograft rejection, and phenotype loss (17). Although many previous studies have reported that ADSCs have appropriate chondrogenic differentiation potential (9, 10), some studies have shown that using bone marrow stromal cells for chondrogenesis is better than using ADSCs (11). In this study, ADSCs were isolated from human adipose tissue and were cultured using HAM as a scaffold for cartilage tissue engineering. Our results indicate that the stromal side of HAM has special features and could function as a suitable biomaterial scaffold for culture and chondrogenic differentiation of ADSCs. We identified ADSCs based on morphology, plastic-adhesion ability, and surface markers according to the International Mesenchymal and Tissue Stem Cell Committee announcement in 2006 (18). Fourth-passaged ADSCs were spindle shaped and were positive for CD105 and CD90 but were negative for CD45 and CD31, similar to the findings reported by De Francesco *et al* and Dominici *et al* (19).

There are certain limitations to the use of cell suspension in cell therapy for cartilage defect. Methods that localize cells to the site of cartilage lesions are less invasive and more attractive for clinical use (20). By intra-articular injection of MSCs, cells adhere to the synovial membrane, and only a small amount of these cells are observed at the site of cartilage defect. By further injection, more cells attach to this site, but the number of cells that adhere to the synovial membrane also increases, thereby increasing the risk of synovial proliferation (21). Therefore, the use of scaffolds for cell delivery to the lesion site would be more appropriate.

HAM is a fetal membrane that was first used by Davis for skin transplantation in 1910 (22). HAM has many advantages that make it a suitable scaffold in tissue engineering such as antimicrobial, antifibrosis, antiscarring, and anti-inflammatory capacities as well as sufficient mechanical properties (23, 24). HAM adheres to the lesion site effortlessly, promotes wound healing, reduces pain, and has low immunogenicity (25). Additionally, it is inexpensive and a waste biomaterial that can be easily processed, cryopreserved, and de-epithelialized (23). Cell adhesion to a scaffold mainly depends on ECM components of the scaffold. HAM comprises epithelium, basement membrane, and an avascular stromal layer (26). Among these, the basement membrane is one of the thickest membranes found in human tissues and is composed of collagen (types III and IV), laminin, fibronectin, and other proteoglycans that are important for cell adhesion and growth. In the avascular stromal layer, collagen types I and III are abundant and have a significant role in maintaining the mechanical properties of the HAM. as well as, in the stromal layer, there is perlecan that is involved maintain the mechanical properties of HAM as well as perlecan that is involved in the binding of growth factors and interaction with ECM and cell adhesion molecules (24, 27). In a previous study, we showed that HAM could be a suitable scaffold for culturing fibroblasts, and we provided an acceptable impermanent skin substitute for skin wounds (12). In the present study, we demonstrated that ADSCs can be effectively cultured and differentiated on HAM while remaining viable; thus, HAM could be used as a suitable scaffold in cell transplantation. Its stromal and epithelial layers have different characteristics. Diaz-prado *et al* cultured human chondrocytes on HAM and reported that these cells grow on the stromal layer better than on the epithelial layer (27). In another study, chondrocytes were cultured on the epithelial layer of intact HAM, epithelial layer of denuded HAM, and stromal layer of denuded HAM. The results showed better growth and penetration of chondrocytes into the stromal layer of denuded HAM and increased expression of collagen type II in this group (16). Chen *et al* (2012) also revealed that culturing and osteogenic differentiation of human dental apical papilla cells on the stromal layer of HAM is more appropriate that those on the epithelial layer (28). However, Yang *et al* indicated that human keratinocytes adhere and grow better on the epithelial layer of denuded HAM, with fibroblasts being seeded on the stromal layer (29). We showed that cultivation and differentiation of ADSCs on the stromal layer of HAM were more appropriate than those on the epithelial layer. ADSCs properly adhered to the stromal layer and integrate into it as well as had a suitable thickness.

In this study, we showed that direct cell differentiation on HAM is better than transferring differentiated cells onto it. Nogami *et al* showed that the rate of expression of collagen type II increases in stem cells cultured on HAM. They mentioned that HAM containing stem cells could be a suitable scaffold for cartilage deficiency (30).

In the three methods used for engineering cartilage tissue, differentiation of ADSCs on the stromal side of HAM showed better results. Compared with scaffold-mediated differentiation, micromass culture showed necrotic cells at the center of micromass; furthermore, the micromass could not attach to both sides of HAM. We previously showed that micromass culture is better than monolayer method for chondrogenic differentiation of ADSCs (31). HAM could be used as a suitable scaffold for cultivation and differentiation of ADSCs without the presence of necrotic cells and can be applied to articular cartilage restoration.

##  Limitation

One of the limitations of this study is the lack of evaluation of collagen type II and aggrecan with immunohistochemistry or RT-PCR.

## 5. Conclusion 

In conclusion, ADSCs have excellent attachment, proliferation, and differentiation capacity on the stromal layer of HAM. Moreover, the direct culture and differentiation of ADSCs on HAM is a more suitable method than the culture of chondrogenic differentiated cells on HAM, and the former method can provide an acceptable engineered cartilage tissue that can be used for cartilage regeneration.

##  Conflict of Interest 

There is no conflict of interest in this study and publication.
